# Preconceptional, Gestational, and Lactational Exposure to an Unconventional Oil and Gas Chemical Mixture Alters Energy Expenditure in Adult Female Mice

**DOI:** 10.3389/fendo.2019.00323

**Published:** 2019-05-22

**Authors:** Victoria D. Balise, Jennifer N. Cornelius-Green, Christopher D. Kassotis, R. Scott Rector, John P. Thyfault, Susan C. Nagel

**Affiliations:** ^1^Department of Obstetrics, Gynecology and Women's Health, University of Missouri, Columbia, MO, United States; ^2^Department of Biological Sciences, University of Missouri, Columbia, MO, United States; ^3^Nicholas School of the Environment, Duke University, Durham, NC, United States; ^4^Department of Nutrition and Health Exercise Physiology, University of Missouri, Columbia, MO, United States; ^5^Medicine-Division of Gastroenterology and Hepatology, University of Missouri, Columbia, MO, United States; ^6^Research Service, Harry S Truman Memorial Veterans Medical Center, Columbia, MO, United States; ^7^Department of Molecular and Integrative Physiology, University of Kansas Medical Center, Kansas City, KS, United States; ^8^Kansas City VA Medical Center, Research Service, Kansas City, MO, United States

**Keywords:** unconventional oil and gas, energy expenditure, endocrine disrupting chemicals, developmental origins of health and disease, hydraulic fracturing, metabolism, metabolic disruptors

## Abstract

Previous studies conducted in our laboratory have found altered adult health outcomes in animals with prenatal exposure to environmentally relevant levels of unconventional oil and gas (UOG) chemicals with endocrine-disrupting activity. This study aimed to examine potential metabolic health outcomes following a preconception, prenatal and postnatal exposure to a mixture of 23 UOG chemicals. Prior to mating and from gestation day 1 to postnatal day 21, C57BL/6J mice were developmentally exposed to a laboratory-created mixture of 23 UOG chemicals in maternal drinking water. Body composition, spontaneous activity, energy expenditure, and glucose tolerance were evaluated in 7-month-old female offspring. Neither body weight nor body composition differed in 7-month female mice. However, females exposed to 1.5 and 150 μg/kg/day UOG mix had lower total and resting energy expenditure within the dark cycle. In the light cycle, the 1,500 μg//kg/day group had lower total energy expenditure and the 1.5 μg/kg/day group had lower resting energy expenditure. Females exposed to the 150 μg/kg/day group had lower spontaneous activity in the dark cycle, and females exposed to the 1,500 μg/kg/day group had lower activity in the light cycle. This study reports for the first time that developmental exposure to a mixture of 23 UOG chemicals alters energy expenditure and spontaneous activity in adult female mice.

## Introduction

Unconventional oil and gas (UOG) extraction combines directional drilling and hydraulic fracturing to liberate oil and gas that was previously inaccessible by traditional drilling methods, including sources of shale gas, coal bed methane, and tight gas. Across the industry, over 1,000 chemicals have been reportedly used in the hydraulic fracturing process. Varying mixtures of these chemicals are combined with millions of gallons of water to fracture underground rock. UOG extraction has been identified as a potential source of EDCs, developmental and reproductive toxicants. We have previously reported that out of 24 UOG chemicals tested, 23 exhibited antagonist activity for one or more of the estrogen, androgen, progesterone, thyroid, and glucocorticoid receptors; and a mixture of 23 of these UOG chemicals exhibited antagonistic activity for all five receptors (1).

UOG activities, including drilling, hydraulic fracturing, and wastewater removal and storage, can contaminate surface and ground water with endocrine-disrupting chemicals (EDCs), defined as exogenous chemicals that can interfere with normal hormone action [(2–6) and reviewed in (7–9)]. We have previously observed an association between endocrine-disrupting activity in surface water and UOG activities. For example, we measured greater antagonistic activities for the estrogen, androgen, progesterone, thyroid, and glucocorticoid receptors immediately downstream of a UOG wastewater disposal facility relative to upstream (4). Our laboratory has reported that prenatal exposure to a laboratory-created mixture of 23 UOG chemicals was associated with altered organ weights, reproductive endpoints, and body weight in adult offspring of gestationally-exposed C57BL/6 mice, suggestive of developmental programming (1, 10, 11). Previous studies on hydraulic fracturing flowback and produced water also support the hypothesis that UOG chemical mixtures can alter fetal development, as developmentally-exposed zebrafish exhibited reduced reproduction, developmental malformations, and developmental toxicity (12, 13). Additionally, a systematic review by Elliot et al. found that 40% of 240 UOG chemicals with publicly-available reproductive and/or developmental toxicity information had been shown to exhibit developmental toxicity (14).

Developmental exposure to EDCs has also been associated with metabolic disease later in life (15), and these chemicals have been termed “metabolic disruptors” (16, 17). Exposure to multiple EDCs, e.g., bisphenol A (BPA), phthalates, dichlorodiphenyltrichloroethane (DDT), and nicotine, among others, has been associated with one or more altered metabolic endpoints, such as obesity, insulin sensitivity, adipose tissue regulation, and lipid disorders [reviewed in (18) and (19)]. UOG chemicals also have the potential to be metabolic disruptors. We have shown that both a 23-UOG mixture and UOG-impacted surface water samples had adipogenic activity *in vitro* (20). Studies in zebrafish have shown that exposure to UOG wastewater resulted in decreased metabolic rates (12, 21). Two studies reported an association between maternal residential proximity to UOG sites and low birth weight infants, while another found an association between maternal residential proximity to UOG sites and increased birth weights (22–24). Both high and low birth weights are associated with later-life development of obesity (25). We have previously demonstrated that female mice prenatally exposed to a mixture of 23 UOG chemicals from gestation day 11 through birth had increased body weights at postnatal days 7, 13, and 21. Body weight and composition can be indicative of energy imbalance.

Taken together, there is limited but suggestive data linking UOG chemicals and altered metabolism. However, no studies have examined the direct of effects of developmental exposure to UOG chemicals and energy expenditure and activity in adulthood. We hypothesized that preconceptional, gestational and lactational exposure to a laboratory-created mixture of UOG chemicals would alter energy balance in adult mice through modulation of energy expenditure. To test this hypothesis, we exposed female C57BL/6 mice to a mixture of 23 UOG chemicals 5 weeks prior to mating, and from gestation day (GD) 1 to postnatal day (PND) 21, and evaluated body composition, energy expenditure, activity, and glucose tolerance in adult offspring.

## Materials and Methods

### Animals

This study was carried out in accordance with the recommendations of the National Research Council's Guide for the Care and Use of Laboratory Animals. The protocol was approved by the University of Missouri Animal Care and Use Committee. C57BL/6J mice (purchased from Jackson Laboratories) were housed in polysulfone cages, in a barrier facility with a 12 h light/dark cycle. Feed (LabDiet 5053: 13% kcal fat, 3.25% kcal sucrose) and acidified water (in glass bottles) were sterilized and provided *ad libitum*.

### Chemical Mixture and Treatment

**Figure 1:**
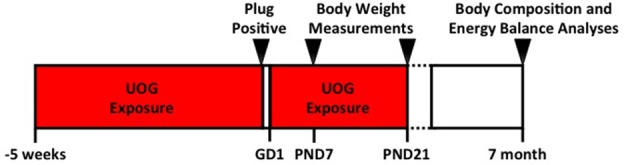
Experimental timeline. Dams (*n* = 14, 9, 11, 8, and 10) that were exposed to the 23 UOG chemical mixture (at concentrations of 0, 0.01, 0.10, 1.0, or 10 μg/mL, respectively) for 5 weeks prior to mating. Chemical exposure was paused while females were mated in order to bypass the window of fertilization, and to avoid consumption of treatment chemicals by the males. Treatment was resumed at gestational day 1 (1 day after presence of copulatory plug) and continued through weaning of the F1 generation at PND 21. Body composition was measured at PND 7, PND 21, and at 7 months of age. Energy balance analyses including energy expenditure, activity, food intake, and glucose tolerance were measured at 7 months of age.

C57BL/6 dams used in this study were 8 months old at initiation of treatment, and 9 months of age when mated. These dams were used in a previous study. Offspring outcomes from the first experiment were reported in Kassotis et al. (1, 11). Each female received the same concentration of chemical mixture that was randomly assigned in the previous study (1). Dams (*n* = 14, 9, 11, 8, and 10) were exposed to the chemical mixture (at concentrations of 0, 0.01, 0.10, 1.0, or 10 μg/mL, respectively) for 5 weeks prior to mating (Figure 1). Chemical exposure was paused while females were mated in order to bypass the window of fertilization, and to avoid consumption of treatment chemicals by the males (Figure 1). Treatment was resumed at gestational day 1 (1 day after presence of copulatory plug) and continued through weaning of the F1 generation at PND 21. “Developmental exposure” will be used throughout the manuscript to describe the inclusive exposure to the dam preconception and GD 1 to PND 21 exposure.

The 23 chemicals were mixed equimass in 200 proof ethanol and added to drinking water such that each individual chemical was present at a concentration of 0.01, 0.10, 1.0, and or 10 μg/mL in a 0.2% ethanol vehicle. Water bottles were changed twice per week to ensure consistent chemical concentrations throughout the dosing period. Water consumption was calculated as the difference in the weight of the water bottle before and after use every time the bottle was changed. Dosages based on weight of the dam and the amount of water consumed were calculated as 1.5, 15, 150, and 1,500 μg/kg/day.

To be included in further analysis, litters had to meet minimum inclusion criteria: Each litter had to have ≥ 3 pups, ≥ 1 male, and ≥ 1 female. After application of inclusion criteria, *n* = 6, 4, 5, 4, and 4 unique litters; and *n* = 9, 11, 9, 10, and 10 individual animals from vehicle, 1.5, 15, 150, and 1,500 μg/kg/day treatment groups, respectively (Supplementary Table 1).

At PND 7, F1 pups were toe clipped and anogenital distance (AGD) was determined by caliper measurement. At PND 21, pups were weaned and rehoused with pups of the same treatment group and sex.

### Animal Rehousing

Female offspring at 6 months of age were transferred to an open-top conventional facility for body composition and metabolic assessments. Mice were allowed to acclimate to the new environment for a month prior to initiation of metabolic testing. This facility was temperature controlled and kept on a 12-h light/dark cycle. In this facility, the experimental animals received non-sterilized feed (LabDiet 5053) and non-acidified, non-sterilized water.

### Body Composition

Body weight was measured at PND 7, PND 21, and 7 months of age. Fat and lean mass were assessed at 7 months of age, using an EchoMRI-900 (EchoMRI, Houston, TX). Fat and lean percentages were calculated by dividing fat or lean mass by body weight.

### Indirect Calorimetry

Energy expenditure via indirect calorimetry, activity, and behavior were measured using the Promethion from Sable Systems Int., (Las Vegas, NV). Oxygen consumption, energy expenditure, and activity were calculated with macros provided by the manufacturer (26).

Total energy expenditure was measured for a 12-h cycle. Resting energy expenditure was extrapolated from the lowest average energy expenditure in a 30-min window within a 12-h cycle and calculated to be representative of the resting energy expenditure for a complete 12-h period. Non-resting energy expenditure was calculated for each 12-h cycle by subtracting 12-h calculated resting energy expenditure from 12-h total energy expenditure (27).

Activity and meters traveled were measured by infrared beams that track movement in horizontal (X and Y plane) and vertical directions (Z plane). Spontaneous activity was defined as activity in the X, Y, and Z directions, ambulatory activity in the X and Y directions, and rearing activity in the Z direction. Meters traveled counted all meters in the X, Y, and Z direction. Food consumption was also measured in this system.

Energy expenditure, activity, and behavior were assessed for a random subset of 7 animals per treatment group on the first day of estrus. Energy expenditure was calculated from measured oxygen consumption using the Kaiyala-Simple equation. Valid data (barring system malfunctions) were collected for *n* = 7, 4, 6, 6, and 6 animals in the vehicle, 1.5, 15, 150, and 1,500 μg/kg/day groups, respectively (Supplementary Table 1). Mice were individually housed in the system's cages for 48 h. The first 24 h were used as an acclimation period, and the second 24 h were analyzed separately as the 12-h light cycle or the 12-h dark cycle. Oxygen consumption, energy expenditure, and activity were calculated with macros provided by the manufacturer (26).

### Glucose Tolerance Test

Glucose tolerance tests were performed only in females in blocks of mice in estrus (*n* = 16/block). Mice were weighed at 1000 h, and fasted from 1000 to 1600 h. A baseline (0 min) blood sample was collected via tail snip at 1600–1630 h, and blood glucose was determined using a glucose monitor (Accu-Chek Aviva Plus). Immediately after the baseline measurement was taken, 250 mg/mL glucose was injected intraperitoneally at 1 mg/kg body weight. Blood glucose concentrations were measured at 30, 60, and 120 min post injection, as described previously (28).

### Statistics

Data were analyzed with a linear mixed model, using SPSS version 32. This model was selected so that litter could be incorporated as a random effect. Treatment and date of measurement (if more than 1 day) were included as fixed effects for body weight, fat mass, lean mass, fat percent, lean percent, food consumption, and activity. For analysis of energy expenditure, body weight and size of litter were also considered as fixed effects. Data were normally distributed or transformed to achieve normality. Results are displayed in all figures as the estimated marginal means, back transformed for presentation if transformation was necessary, except for Supplemental Figure 1C. Differences between vehicle and treatment groups were analyzed using Fisher's Least Significant Difference tests, with 95% confidence intervals. The percent dams that delivered (Supplemental Figure 1C) was analyzed by Fisher's exact test. All tests were compared to vehicle.

## Results

### Maternal and Birth Outcomes

The body weights of pregnant dams were measured in order to monitor health and calculate treatment dosage. The body weights of the dams exposed to the UOG chemical mixture did not differ from those of dams exposed to the vehicle at gestation day 0 after 5 weeks of treatment (Supplementary Figure 1A). Treatment did not alter dam body weight or water consumption (Supplementary Figures 1A,B). The percentage of dams per group that delivered tended to be decreased in the 150 and 1,500 μg/kg/day groups (p<0.20) (Supplementary Figure 1C). The number of live pups per litter did not differ relative to vehicle (Supplementary Figure 1D).

### Offspring Body Composition

**Figure 2:**
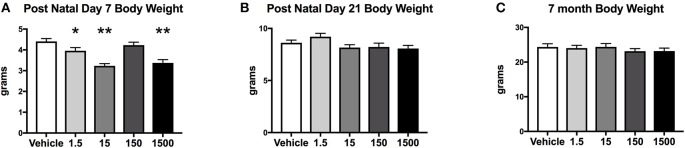
Body weights of offspring. Estimated marginal means (**±**) SEM of body weight at post-natal day 7 **(A)**, post-natal day 21 **(B)**, and at 7 months of age **(C)**. *p < 0.05 relative to vehicle **p < 0.0125 relative to vehicle (*n* = 9, 11, 9, 10, 10 respectively for vehicle, 1.5, 15, 150, and 1,500 μg/kg/day treatment groups). Models included covariates: litter, date body weight was taken and litter size.

Developmental exposure to the UOG chemical mixture altered the body weights of female offspring at PND 7. Body weight at PND 7 in F1 females developmentally exposed to the UOG chemical mixture was 10–26% lower in the 1.5, 15, and 1,500 μg/kg/day treatment groups relative to vehicle (Figure 2A). At PND 21 and at 7 months of age, these females no longer displayed differences in body weight relative to vehicle (Figures 2B,C). Fat mass, percent fat mass, lean mass, and percent lean mass at 7 months of age also did not differ relative to vehicle (Supplemental Figure 2).

### Offspring Energy Expenditure

**Figure 3:**
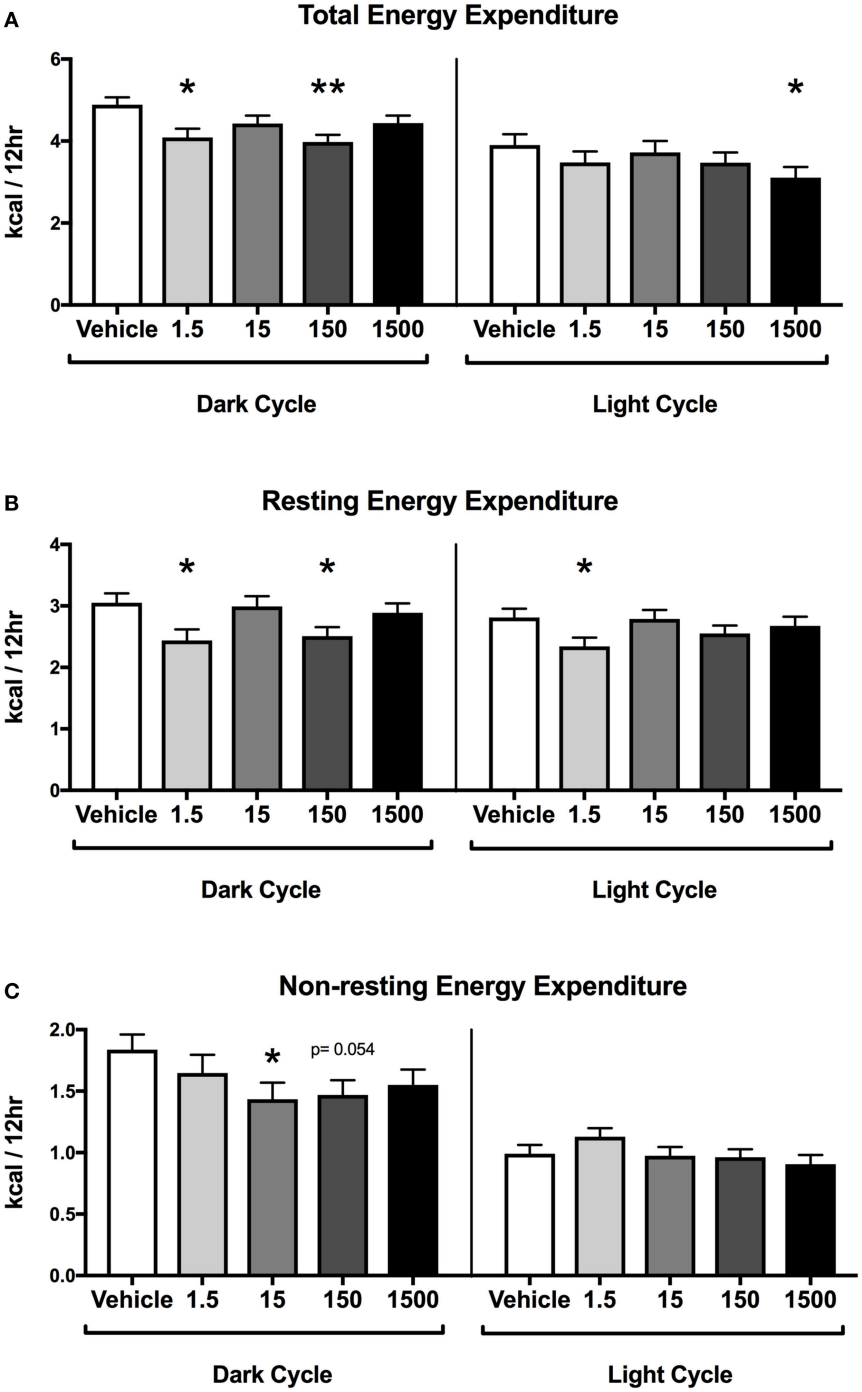
Energy Expenditure in Female Offspring at 7 months of age. Estimated marginal means (**±**) SEM in 12-h average increments of total energy expenditure **(A)**, resting energy expenditure **(B)**, non-resting expenditure **(C)** (*n* = 7, 4, 6, 6, 5 respectively for vehicle, 1.5, 15, 150, and 1,500 μg/kg/day treatment groups). *p < 0.05 relative to vehicle **p < 0.0125 relative to vehicle. Models included covariates: litter, date of recording, litter size, and body weight.

Developmental exposure to the UOG chemical mixture was associated with altered energy expenditure in the dark cycle in females. After 24 h of acclimation, energy expenditure was assessed for the final 24 h. Energy expenditure data were divided into 12-h light and dark cycles for analysis. In the dark cycle, total energy expenditure was 16 and 19% lower and resting energy expenditure was 20 and 18% lower in the 1.5 and 150 μg/kg/day treatment groups respectively (Figures 3A,B). Non-resting energy expenditure tended to be 22 and 20% lower in the 15 (*p* = 0.054) and 150 (*p* = 0.054) μg/kg/day treatment groups relative to vehicle (Figure 3C).

In the light cycle, total energy expenditure was 20% lower in the 1,500 μg/kg/day treatment group relative to vehicle (Figure 3A). Resting energy expenditure was 17% lower in the 1.5 μg/kg/day group relative to vehicle, while non-resting energy expenditure was not altered relative to vehicle in any treatment group (Figures 3B,C).

### Offspring Activity

**Figure 4:**
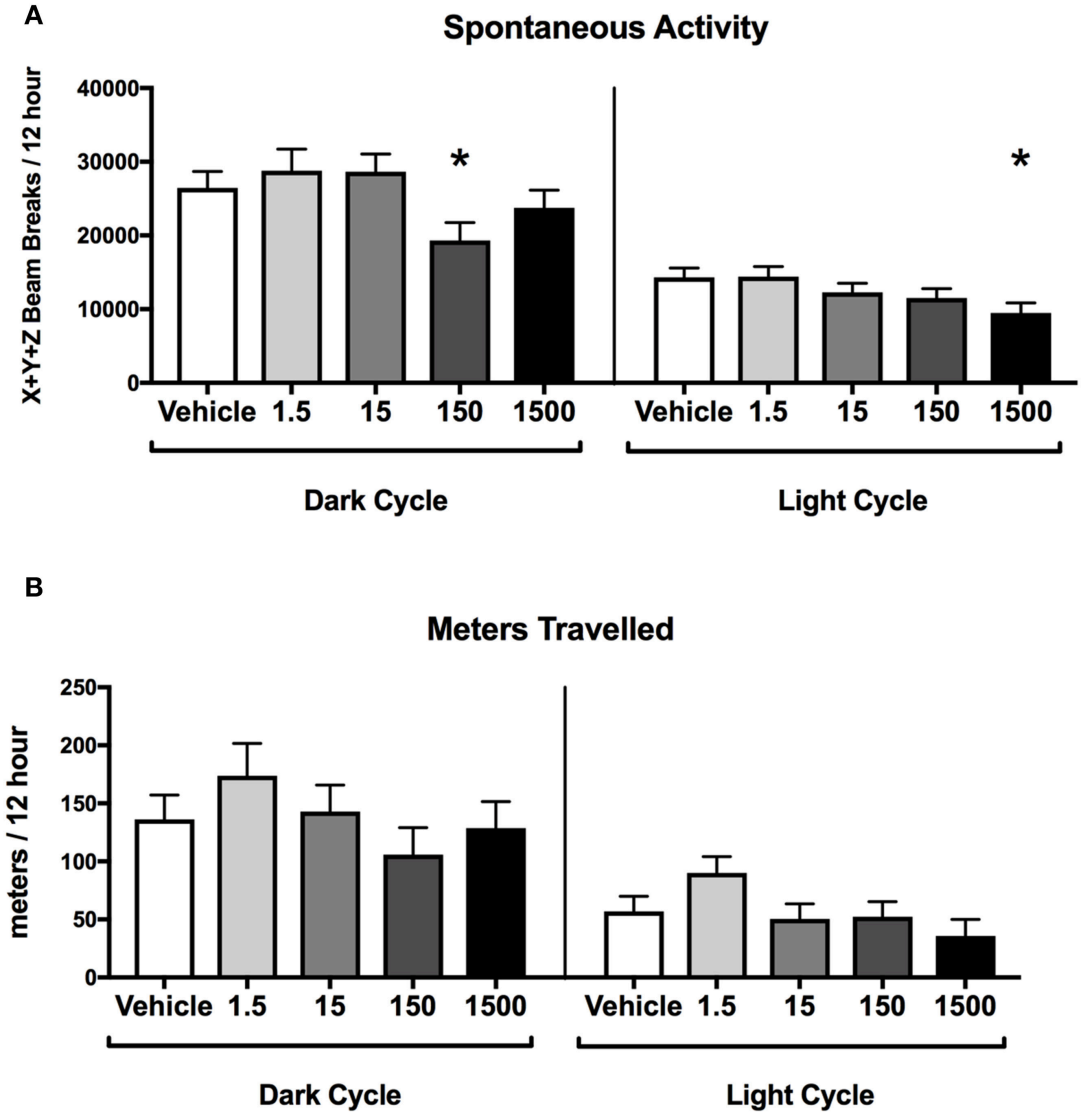
Activity of female offspring at 7 months of age. Estimated marginal means (**±**) SEM in 12-h average increments of total spontaneous activity **(A)**, and meters travelled **(B)** (*n* = 7, 4, 6, 6, 5 respectively for vehicle, 1.5, 15, 150, and 1,500 μg/kg/day treatment groups). *p < 0.05 relative to vehicle. Models included covariates: litter and date of recording.

Developmental exposure to the UOG chemical mixture was associated with altered spontaneous activity in both the light and dark cycles in females. In the dark cycle, spontaneous activity was 27% lower in the 150 μg/kg/day treatment group relative to vehicle (Figure 4A). In the light cycle, spontaneous activity was 34% lower in the 1,500 μg/kg/day treatment group relative to vehicle (Figure 4B). No differences were detected in ambulatory, rearing activity, or meters traveled for any treatment group relative to vehicle in the light or the dark cycles (Supplementary Figure 3).

### Offspring Glucose Homeostasis

Glucose tolerance tests were performed at 7 months of age on the day of estrus. No differences were detected in basal glucose levels, glucose levels at subsequent time points using basal glucose as a baseline, or in area under the curve for any treatment groups in females (Supplementary Figure 4).

### Offspring Food Consumption

**Figure 5:**
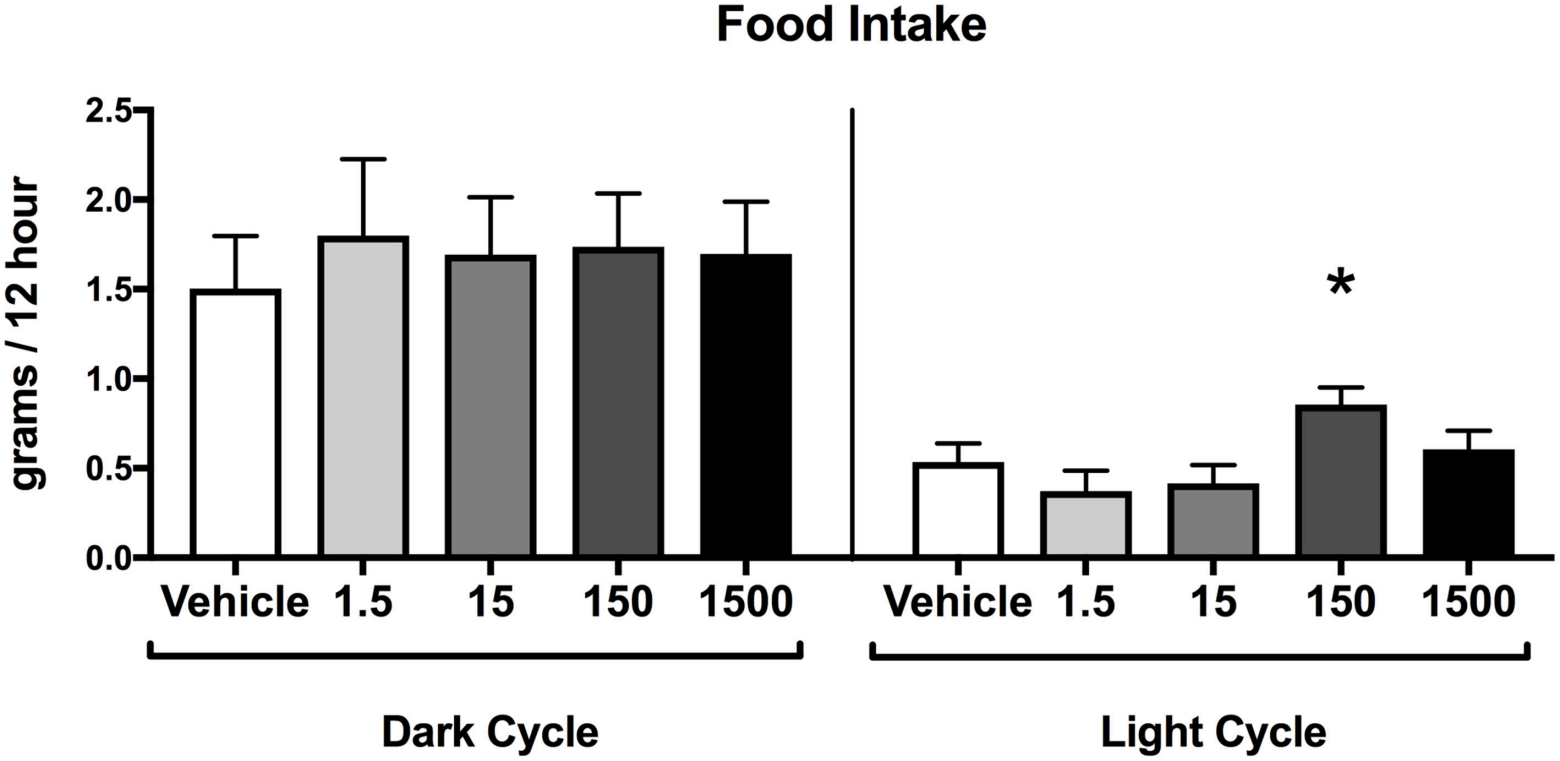
Food consumption in female offspring at 7 months of age. Estimated marginal means (**±**) SEM of food intake at 7 months of age in 12 h increments of both light and dark cycle (*n* = 7, 4, 6, 6, 5 respectively for vehicle, 1.5, 15, 150, and 1,500 μg/kg/day treatment groups). *p < 0.05 relative to vehicle. Models included date of recording as a covariate.

Food consumption during the dark cycle did not differ between vehicle and treatment groups in 7-month-old female offspring. However, food consumption during the light cycle increased by 60% in the 150 μg/kg/day treatment group relative to vehicle (Figure 5), but no differences were detected in the other treatment groups when compared to vehicle.

## Discussion

We report for the first time that developmental exposure to a mixture of 23 oil and gas chemicals altered adult energy expenditure in 7-month-old female mice, particularly in the dark cycle when mice are more active. Mice in the 15 μg/kg/day group had a lower non-resting energy expenditure. Females in the 1.5 and 150 μg/kg/day groups had lower total and resting energy expenditure within the dark cycle, and the 150 μg/kg/day group had lower spontaneous activity and tended to have lower non-resting energy expenditure in the dark cycle. This decrease in energy expenditure did not result in altered body weight or body composition at 7 months of age. This study supports the hypothesis that developmental exposure to EDCs can contribute to the programming of energy expenditure and activity in adulthood.

Hormones are essential in regulating metabolism throughout development and programming metabolic function in adulthood, and developmental exposure to EDCs has been reported to alter body composition, energy expenditure, activity, glucose homeostasis and adipogenesis (29–32). Developmental exposure to EDCs, including BPA, lead, arsenic, diethylstilbestrol (DES), and perfluorooctanoic acid (PFOA) has been associated with altered metabolism (19, 29–39). While these EDCs are reported to disrupt one or more hormone receptors, including estrogen, androgen, progesterone, glucocorticoid, thyroid hormone, and others, they have all been reported to agonize the estrogen receptor (33, 35–37, 39–41). Developmental exposure to estrogen receptor agonists from preconception to weaning is associated with increased energy expenditure and spontaneous activity in female Agouti (lead and BPA) and C57BL/6JxFVB (BPA) mice (29, 34, 38). These effects may be strain specific as no difference in energy expenditure was seen in CD-1 and California mice (42–46).

The role of androgens in metabolic dysfunction are well-appreciated, though are not likely to play a role in the observed effects herein. Acute androgen exposure is generally considered anti-adipogenic and anti-androgen exposure is adipogenic using *in vitro* or *in vivo* models (47, 48). However, dissimilar effects can be observed in specific cases. Women, such as those with polycystic ovarian syndrome (PCOS), have increased serum androgens and suffer increased visceral white adipose tissue deposition (47), potentially mediated by reduced insulin sensitivity (48, 49). These effects also appear reversed with developmental androgen exposure. For example, prenatal exposure to androgen results in metabolic dysfunction in adult female rodents and monkeys, including increased body weight, adiposity, insulin, serum lipids profiles, and decreased energy expenditure (1, 49–51).

We have previously reported that the UOG mix has antagonist activity for estrogen, androgen, glucocorticoid, progesterone, and thyroid hormone receptors suggesting the UOG mix may antagonize one or more of these receptors during development to alter metabolic endpoints in adulthood (10). In this study, females exposed to the 1.5 and 150 μg/kg/day 23-UOG mix had lower total and resting energy expenditure and 15 μg/kg/day had lower non-resting energy expenditure in the dark cycle. Further, females developmentally exposed to the 15 and 150 μg/kg/day 23-UOG mix had lower spontaneous activity. These effects are the opposite of the increased energy expenditure and spontaneous activity after developmental exposure to estrogen receptor agonists (34, 38); thus, the UOG mixture may have programmed reduced energy expenditure and spontaneous activity at 7 months of age due to estrogen receptor antagonism during development. While there is little information on the developmental effects of estrogen receptor antagonists and adult energy expenditure, depletion of estrogen receptor activity (estrogen receptor-alpha knockout and g-protein coupled estrogen receptor knockout) has been associated with lower total energy expenditure suggesting estrogen receptor activity may modulate development of energy homeostasis in adulthood (50, 51). Androgenic effects during gestation could elicit some of the effects reported herein; however, since the UOG mix contains anti-androgenic activity rather than agonist activity, the effects observed in the current study in 7-month-old female mice, do not appear to be mediated through AR (1, 10). Taken together, while the lower energy expenditure and activity seen in the current study is consistent with antiestrogenic activity in the 23-UOG mixture, future studies are needed to delineate the exact developmental receptor pathways modulated by the 23-UOG mixture during development that alter adult energy expenditure.

In the current study, we expanded the exposure window from our prior work using a prenatal exposure (GD 11–18) to combine pre-conception, prenatal, and lactational exposure to assess impacts of adult maternal exposure prior to fertilization and to bracket fetal development from GD 1 to PND 21. Exposure during GD 1–11 covers development of the placenta, pancreas, and liver, and maternal high fat diet during this period has been shown to cause adverse metabolic outcomes in offspring (52). Also, a prolonged exposure through PND 21 includes development of the brain including expression of neurotransmitters and their receptors (53). The brain is a key modulator of energy balance regulating food intake, energy expenditure, and insulin secretion (54). UOG chemicals are cleared from the body within hours, so the exposure window for this study did not cover the time of mating to avoid male exposure as it has been shown male sperm can effect the epigenetics of offspring leading to obesity (55). Exposure started at GD 1, which is 24–36 h after mating depending on exactly when copulation occurred. This exposure paradigm is largely after the major wave of the zygotic activation phase between ~24 and 36 h, when the embryo is becoming transcriptionally active, which will result in some heterogeneity of exposure depending on when copulation occurred (56, 57). Epigenetic remodeling occurs during this developmental phase and experiments should specifically target this phase for exposure to determine if UOG chemical exposure alters epigenetic reprogramming (56, 58). Future work is needed to systematically assess the impacts of UOG exposure on the epigenetics of offspring and the unique impacts of different exposure windows.

In the current study, we report that a combined pre-conceptional, prenatal, and lactational exposure from GD 1 to PND 21 to a mixture of 23 UOG chemicals was associated with decreased body weights at PND 7 in females. Previously our lab has shown that a prenatal exposure from GD 11 to GD 18 to the same 23-UOG mixture resulted in the opposite–increased body weight at PND 7 and 21 (11). This may be due in part to the different exposure windows as developmental exposure to environmental chemicals can have quantitatively and qualitatively different effects depending on the exposure windows (59–61). Alternatively, decreased body weight at PND 7 in the current study could have been a transient acute effect from lactational exposure to the 23-UOG mixture or a result of altered maternal behavior as EDCs have been shown to disrupt maternal behavior (62–64).

Many factors contribute to energy balance, body composition, and body mass regulation. In this study, pre- and post-natal exposure to the 23-UOG mixture decreased total and resting energy expenditure in some UOG mix groups, but this did not result in altered body weight, lean mass, or fat mass in 7-month-old females. Although one would typically expect higher body mass or fat mass to track with lower energy expenditure, this is not always the case. For example, Wan et al. also found that AKT knockout mice displayed an increase in energy expenditure compared to control mice matched for body mass (65). A limitation of indirect calorimetry is that it is taken at one point in time and does not represent energy metabolism throughout the lifespan of the animal. It is possible that the lower energy expenditure measured in the 23-UOG mixture may have led to greater body mass if mice were aged longer-a question directly assessed in a companion paper in this journal, Balise et al. (submitted). In addition, although efforts were made to reduce any stress caused by the indirect calorimetry cages by providing an acclimation period and using the same bedding as home cages, it is possible that a change to a new environment impacted control mice and 23 UOG exposed mice differently during the defined period of time. Energy homeostasis is maintained with different compensating mechanisms such as differences in digestion, skeletal muscle metabolism, adipose storage, or fecal deposition. In addition, although we carefully measured food intake at defined periods of time, it is possible that small reductions in food intake allowed 23-UOG mixture treated animals to maintain normal body mass despite reduced energy expenditure. Future long-term studies can be conducted to determine if these significant decrements in energy expenditure have long term ramifications for body mass and metabolic health. At this age, an impact on body weight might not be seen unless the system is challenged beyond compensatory mechanisms. For example, a high-fat diet or western style diet challenge has revealed underlying metabolic programming following developmental exposure to other EDCs, such as DEHP, atrazine, and BPA (66–68). Further studies challenging these animals with a high fat high sugar diet might reveal underlying metabolic differences by challenging the homeostatic mechanisms that regulate metabolism (see Balise et al., submitted).

Overall, we have reported that the 23-UOG mixture can alter developmental programming and result in altered energy expenditure and activity of 7-month-old females. The results shown thus far highlight the need for additional research on metabolic health effects in humans and animals in drilling-dense regions. More studies should be aimed at understanding exposure to UOG and other environmental chemicals on metabolic health outcomes (69).

## Ethics Statement

This study was carried out in accordance with the recommendations National Research Council's Guide for the Care and Use of Laboratory Animals. The protocol was approved by the University of Missouri Animal Care and Use Committee.

## Author Contributions

VB and JC-G performed animal experiments. VB analyzed the data. SN secured funding, directed experiments and assisted in data analysis and interpretation. All authors have contributed to the design of experiments, interpretation of the data, and writing the manuscript.

### Conflict of Interest Statement

The authors declare that the research was conducted in the absence of any commercial or financial relationships that could be construed as a potential conflict of interest.
