# Supplementary material for: Preconceptional, Gestational, and Lactational Exposure to an Unconventional Oil and Gas Chemical Mixture Alters Energy Expenditure in Adult Female Mice
Source: Front Endocrinol (Lausanne). 2019 May 22;10:323. doi: 10.3389/fendo.2019.00323 (PMC6540741; doi:10.3389/fendo.2019.00323)
Supplement: Supplementary file 1 [file Data_Sheet_1.docx]

**Supplementary Table 1**. Sample size (N) for dams, F1 pups, unique litters, and distribution of animals within unique litters.

|  | Dose (µg/kg/day) | | | | |
| --- | --- | --- | --- | --- | --- |
|  | Vehicle | 1.5 | 15 | 150 | 1500 |
| Dams that were mated | 14 | 9 | 11 | 9 | 10 |
| Dams that plugged | 14 | 9 | 11 | 9 | 10 |
| Dams that delivered | 11 | 6 | 9 | 5 | 6 |
| Litters cannibalized | 3 | 1 | 0 | 0 | 1 |
| Dams with pups at PND7 | 8 | 5 | 9 | 5 | 5 |
| Litter used in separate study | 1 | 0 | 3 | 0 | 0 |
| Litters that did not meet inclusion criteria* | 1 | 1 | 1 | 1 | 1 |
| # of litters used | 6 | 4 | 5 | 4 | 4 |
| N for female body composition | 10 | 11 | 9 | 10 | 10 |
| N/litter for body composition | 2, 1, 1, 3, 1, 2 | 2, 6, 1, 2 | 2, 3, 1, 2, 1 | 1, 4, 1, 4 | 2, 2, 2, 4 |
| N for female Indirect Calorimetry | 7 | 4 | 6 | 6 | 5 |
| N/litter Indirect Calorimetry | 2, 1, 1, 2, 1 | 2, 1, 1 | 1, 1, 1, 2, 1 | 1, 3, 2 | 1, 1, 1, 1, 1 |

***** Litter inclusion criteria: ≥ 1 male/litter, ≥ 1 female/litter, and ≥ 3 mice/litter

**Supplemental Figure 1. Maternal Health.** Health of dams shown through estimated marginal means (+/-) SEM of body weight at gestational day zero before exposure (A), water consumption throughout exposure (B), percent of dams that were shown to be plug positive and delivered, data analyzed by Fisher’s Exact Test (C), and total number of pups at delivery (D) (n= 14, 9, 11, 8, 10 respectively for vehicle, 1.5, 15, 150, and 1500 µg/kg/day treatment groups).

**Supplemental Figure 2. Body Composition of offspring.** Estimated marginal means (+/-) SEM of body composition taken at 7 months for fat mass (A), percent fat (B), lean mass (C), and percent lean (D) (female n= 9, 11, 9, 10, 10 respectively for vehicle, 1.5, 15, 150, and 1500 µg/kg/day treatment groups, male n=8 for vehicle, 1,5 and 150 µg/kg/day treatment groups). * p<0.05 relative to vehicle ** p<0.0125 relative to vehicle. Models included covariates: litter, date body weight was taken and litter size.

******

**Supplementary Figure 3. Ambient and Rearing Activity of Female Offspring at 7 months of age.** Estimated marginal means (+/-) SEM in 12-hour average increments of ambient activity (A), and rearing activity (n= 7, 4, 6, 6, 5 respectively for vehicle, 1.5, 15, 150, and 1500 µg/kg/day treatment groups). Models included covariates: litter and date of recording.

**Supplementary Figure 4. Glucose Tolerance Test in Female Adult Offspring.** Estimated marginal means (+/-) SEM of basal blood glucose levels (A) at time point 0, 30, 60 and 120 minutes after glucose injection relative to basal blood glucose level (B), area under the curve of glucose tolerance test (C) (n= 9, 11, 9, 10, 10 respectively for vehicle, 1.5, 15, 150, and 1500 µg/kg/day treatment groups).
